# IL-6 potentiates BMP-2-induced osteogenesis and adipogenesis via two different BMPR1A-mediated pathways

**DOI:** 10.1038/s41419-017-0126-0

**Published:** 2018-02-02

**Authors:** Ru-Lin Huang, Yangbai Sun, Chia-Kang Ho, Kai Liu, Qi-Qun Tang, Yun Xie, Qingfeng Li

**Affiliations:** 1 Department of Plastic and Reconstructive Surgery, Shanghai Ninth People’s Hospital, Shanghai Jiao Tong University School of Medicine, 639 Zhizaoju Road Shanghai, 200011 China; 2 Key Laboratory of Metabolism and Molecular Medicine, the Ministry of Education, Department of Biochemistry and Molecular Biology, Fudan University Shanghai Medical College, Shanghai, 200032 China

## Abstract

Recombinant human bone morphogenetic protein-2 (rhBMP-2) is widely used in the clinic for bone defect reconstruction because of its powerful osteoinductive capacity. However, commercially available rhBMP-2 requires a high concentration in the clinical setting for consistent bone formation. A high dose of rhBMP-2 induces a promising bone formation yield but also leads to inflammation-related events, deteriorated bone quality, and fatty tissue formation. We hypothesize that the seemingly contradictory phenomenon of coformation of new bone and excessive adipose tissue in rhBMP-2-induced bone voids may be associated with interleukin-6 (IL-6), which is significantly elevated after application of rhBMP-2/absorbable collagen sponge (rhBMP-2/ACS). Here, we show that IL-6 injection enhances new bone regeneration and induces excessive adipose tissue formation in an rhBMP-2/ACS-induced ectopic bone formation model in rats. In vitro data further show that IL-6 and its soluble receptor sIL-6R synergistically augment rhBMP-2-induced osteogenic and adipogenic differentiation of human BMSCs (hBMSCs) by promoting cell surface translocation of BMPR1A and then amplifying BMPR1A-mediated BMP/Smad and p38 MAPK pathways, respectively. Our study suggests elevated IL-6 may be responsible for coformation of new bone and excessive adipose tissue in rhBMP-2-induced bone voids.

## Introduction

Recombinant human bone morphogenetic protein-2 (rhBMP-2) is a powerful osteoinductive protein in terms of its ability to recruit mesenchymal stem (or precursor) cells of different tissue origin and induce these cells to become osteoblastic cells. Indeed, rhBMP-2 was granted U.S. Food and Drug Administration approval in 2002 as a bone graft substitute for use in spine fusion, open tibial fractures, sinus augmentations, and alveolar ridge defects^1^. Because of its short half-life and poor delivery system, commercially available rhBMP-2 (INFUSE; Medtronic, TN, USA), which is impregnated in an absorbable collagen sponge (ACS), requires a high concentration in the clinical setting for consistent bone formation. The rhBMP-2 concentration of INFUSE is set at 1.5 to 2.0 mg/mL (ref.^2^), which greatly exceeds the concentrations required in nonhuman primates (0.75 to 2.0 mg/mL) and rodents (0.02 to 0.4 mg/mL) as well as human endogenous concentrations (18.8 to 22 pg/mL)^3^. While high doses of rhBMP-2 lead to a promising bone formation yield, these doses do not benefit clinical outcomes and cause a significant increase in complication rates^4–6^. Thus, increasing concerns regarding the efficacy and safety profile of high doses of rhBMP-2 in clinical practice remain a challenging clinical problem^7–9^.

Among the reported complications associated with the application of high-dose rhBMP-2, inflammation-related events, deteriorated bone quality, and fatty tissue formation have attracted attention. Clinical and experimental studies have revealed that high doses of rhBMP-2 induce an exaggerated inflammatory response, characterized by both the recruitment of inflammatory cells to the implantation site and a significant elevation of cytokines in sera, resulting in impaired bone regeneration^10–12^. Clinical observations have determined that the quality of rhBMP-2-induced bone tissue appears to be poorer than that of native bone, exhibiting a significantly lower density^13^, cyst-like bone void formation^14^, and a larger area of fatty bone marrow formation. Interestingly, these bone voids, which are diagnosed as seroma, are typically observed in both newly formed bone tissue and excessive adipose tissue after the application of rhBMP-2^5,15–17^. This phenomenon remains a matter of controversy and requires further studies to determine the underlying molecular mechanism.

Osteoblasts and adipocytes originate from common precursor cells, and a mutually inhibitory relationship exists between osteogenic and adipogenic lineage commitment and the differentiation of mesenchymal stem cells (MSCs)^18^. However, although BMP-2 is an effective osteoinductor, it also induces adipogenesis in MSC cultures^19,20^. In clinical conditions, the interaction between rhBMP-2 and inflammatory cytokines, which show elevated levels in the exaggerated inflammatory environment, makes the commitment and differentiation of human bone marrow mesenchymal stem cells (hBMSCs) more complicated. Among the elevated inflammatory mediators, tumor necrosis factor (TNF)-α and interleukin (IL)-1β play an inhibitory role in the rhBMP-2-induced osteogenic differentiation of BMSCs^11,21,22^. However, IL-6 seems to be an exception, showing a synergetic effect with rhBMP-2 to enhance bone regeneration in an animal model^23^. IL-6 is a highly pleiotropic cytokine, and its effects on bone and adipose metabolism are controversial and unresolved^24^. In bone metabolism, IL-6 promotes the osteogenic differentiation of preosteoblasts and adipose stem cells^25^. In contrast, IL-6 also negatively regulates osteoblastic differentiation in MC3T3-E1 cells^26^ and is responsible for the defective osteogenesis of osteoporotic BMSCs^27^. In adipose metabolism, IL-6 has a catabolic effect on adipogenesis, and an elevated IL-6 level impairs human subcutaneous adipogenesis^28^. IL-6-deficient mice develop obesity and revert to normal once treated with IL-6^29^. However, numerous studies have shown that IL-6 levels are greatly elevated in obese humans and correlate positively with obesity and waist circumference^30^.

Given the involvement of IL-6 in BMP-2-induced adipo-osteogenesis, we hypothesized that the coexistence of new bone and adipose tissue after the application of high-dose rhBMP-2 may be related to the elevated IL-6 level in the exaggerated inflammatory environment to some extent and sought to further define the relationship of BMPR1A-mediated downstream pathways and the regulation of the adipo-osteogenic differentiation of hBMSCs. To confirm this hypothesis, we established a cell model and an animal model for investigating the underlying role of IL-6 in the controversial phenomenon observed after clinical application of rhBMP-2/ACS. We found that IL-6 potentiates rhBMP-2-induced osteogenesis and adipogenesis by promoting the cell surface translocation of BMPR1A and stimulating two distinct downstream pathways.

## Methods and materials

### Reagents, plasmids, and animals

Purified rhBMP-2, IL-6, and sIL-6R were provided by R&D Systems (Minneapolis, MN, USA). Monensin, dorsomorphin homolog 1 (DMH1), naphthol AS-BI alkaline solution, and phalloidin were purchased from Sigma-Aldrich Co. LLC. (St. Louis, MO, USA). Rabbit anti-Smad1 antibody, rabbit anti-BMPR1A antibody, mouse anti-BMPR2 antibody, rabbit anti-CCAAT enhancer-binding protein-α (C/EBPα) antibody and rabbit anti-peroxisome proliferator-activated receptor gamma (PPARγ) antibody were obtained from Abcam (Cambridge, MA, USA). Anti-phospho-Smad1/5/8 antibody, anti-phospho-p38 antibody, anti-p38 antibody, anti-Runx2 antibody, anti-GAPDH antibody, anti-β-actin antibody, and anti-fade reagent were provided by Cell Signaling Technology, Inc. (Danvers, MA, USA). Rabbit anti-BMPR1B antibody, Alexa Fluor-488-conjugated secondary antibody, and TRIzol reagent were obtained from Invitrogen (Carlsbad, CA, USA). FuGENE®HD transfection reagent was provided by Promega BioSystems (Sunnyvale, CA, USA). Smad1 shRNA plasmids were obtained from Santa Cruz Biotechnology (Dallas, TX, USA). Human MSC osteogenic and ADM were purchased from Cyagen Biosciece (Guangzhou, China). Lewis rats were provided by Shanghai Experimental Animal Center China.

### Cell harvest and culture

Human bone marrow was aspirated from the iliac crest of six healthy male and female donors aged 23–46 years, following approval by the Institutional Review Board (IRB) at our institution. Written informed consent was obtained from all patients. The hBMSCs were established as previously described^23,31^. The cells were cultured in Dulbecco’s modified Eagle’s medium (DMEM) supplemented with 10% fetal bovine serum (FBS; Gibco, CA, USA). The culture medium was changed every 3 days. Only BMSCs from early passages (2-4) were used in our experiments.

### Osteogenic and adipogenic differentiation of hBMSCs

The hBMSCs were plated at a low density and cultured in DMEM containing 10% FBS with or without rhBMP-2, IL-6, sIL-6R, monensin, or DMH1 for 3 days. Then, for osteogenic differentiation, the postconfluent cells were grown in human MSC osteogenic differentiation medium (ODM). For adipogenic differentiation, the postconfluent cells were grown in human MSC adipogenic differentiation medium (ADM) A for 3 days and then ADM B for an additional day. After 2–3 cycles, the hBMSCs were treated with ADM B for an additional 6 days.

To quantitatively measure alkaline phosphatase (ALP) activity, the cells were exposed to ODM for 3 days. Then, the cells were lysed, and the cellular ALP activity was measured using an Alkaline Phosphatase Detection Kit (Nanjing Jiancheng Bioengineering Institute, China). The amount of ALP in the cells was normalized against the total protein content. For ALP staining, the cells were maintained in ODM for 6 days. Then, the cells were fixed in 4% paraformaldehyde and stained with naphthol AS-BI alkaline solution to visualize ALP activity. For Alizarin Red S (ARS) staining, the cells were cultured in ODM for 21 days and stained with a 40 mM ARS solution to visualize the matrix calcium deposition. Then, Alizarin was resolubilized to perform a quantitative analysis using spectrophotometry. For Oil Red O (ORO) staining, the cells were induced using adipogenic differentiation for three cycles and stained with an ORO staining solution. Then, the ORO stain was extracted using isopropanol and quantitatively analyzed by spectrophotometry.

### RNA extraction, reverse transcription PCR (RT-PCR), and quantitative real-time PCR analysis

After treatment as described in the relevant results section, the cells were washed with phosphate-buffered saline (PBS) and lysed with the TRIzol reagent according to the manufacturer’s protocol. Then, 2 μg of total RNA was used for reverse transcription, and the product was analyzed by RT-PCR or real-time PCR. RT-PCR was performed using an RT-PCR kit (Takara, China) according to the manufacturer’s instructions. The levels of osteogenic genes were quantified with an ABI 7500 Real-Time PCR System. PCR primer pairs were designed based on the sequences of different exons of the corresponding genes (Table 1). All real-time PCR amplifications were performed with an initial denaturation at 95 °C for 30 s, followed by 40 cycles at 95 °C for 5 s and 60 °C for 34 s, and a melting curve analysis was performed at 95 °C for 15 s and 60 °C for 60 s.

**Table 1:** List of oligonucleotides used for quantitative real-time PCR and RT-PCR

Target gene	Sequence	Reference
*OCN*	F: 5′-ATGAGAGCCCTCACACTCCT-3′	NM_199173.4
	R: 5′-CTTGGACACAAAGGCTGCAC-3′	
*OPN*	F: 5′-TAGGCATCACCTGTGCCATAC-3′	NM_000582.2
	R: 5′-TACTTGGAAGGGTCTGTGGGG-3′	
*ALP*	F: 5′-ATACCTGGGATTTCCGCCTC-3′	NM_031313.2
	R: 5′-GGGTTCTCCTCCTCAACTGG-3′	
*aP2*	F: 5′-GAAGCTTGCAGCTCATGACA -3′	NM_000134.3
	R: 5′-CCCCTGAGTTCAGTTCCGTC-3′	
*LPL*	F: 5′-CGAGCGCTCCATTCATCTCT-3′	NM_000237.2
	R: 5′-CCAGATTGTTGCAGCGGTTC-3′	
*PPARγ*	F: 5′-CCGTGGCCGCAGATTTGA-3′	NM_001285879.1
	R: 5′-AGATCCACGGAGCTGATCCC-3′	
*Cyclin D1*	F: 5′-AAAGAATTTGCACCCCGCTG-3′	NM_053056.2
	R: 5′-GACAGACAAAGCGTCCCTCA-3′	
Cyclin E1	F: 5′-GACGGGGAGCTCAAAACTGA-3′	NM_001238.3
	R: 5′-GGGGAGAGGAGAAGCCCTAT-3′	
*Cyclin B1*	F: 5′-ACCGAATCCCTAGTCCCCC-3′	NM_031966.3
	R: 5′-ACAAAACCAAAATGAAAACTGGCT-3′	
*GAPDH*	F: 5′-AATGGGCAGCCGTTAGGAAA-3′	NM_001256799.2
	R: 5′-GCGCCCAATACGACCAAATC-3′	

*F* forward primer, *R* reverse primer

### Immunofluorescence staining

After treatment as described in the relevant results section, the cells were washed with PBS and then fixed with 1% paraformaldehyde for 30 min. To measure total cellular antigen expression, the cell aliquots were permeabilized using 0.1% Triton X-100 for 15 min at room temperature. The cells were then blocked with 5% goat serum for 60 min. The cells were subsequently immunostained with rabbit anti-Smad1 antibody (1:100), anti-BMPR1A antibody (1:50), rabbit anti-BMPR1B antibody (1:50), or mouse anti-BMPR2 antibody (1:100), followed by incubation with goat anti-rabbit or goat anti-mouse Alexa Fluor-488-conjugated secondary antibody (1:200) and phalloidin. Prior to examination, the samples were covered with anti-fade reagent.

### Transient transfection

All transient transfections were performed using FuGENE®HD transfection reagent. The total amounts of transfected plasmid were equalized across the groups by the addition of an empty vector. For each transfection, hBMSCs were separately transfected with a plasmid expressing Smad1 shRNA to block the intracellular BMP/Smad signal pathway.

### Cell surface biotinylation

Cell surface biotinylation was performed using an EZ-Link™ Sulfo-NHS-SS-Biotinylation Kit (Pierce, IL, USA). Briefly, after treatment, the cells were placed on ice and washed twice with ice-cold PBS, followed by incubation with freshly prepared Sulfo-NHS-SS-Biotin in PBS for 10 min in ice. Biotinylation was terminated by washing the cells twice with PBS. The cells were then lysed with 1 mL of PBS containing 1% Triton X-100 and an EDTA-free protease inhibitor cocktail. Next, avidin-agarose beads were added to 900 μL of lysate and rotated overnight at 4 °C. The remaining 100 mL was used for total protein assessment. The biotin-avidin agarose complexes were subsequently harvested by centrifugation and washed three times with lysis buffer. The beads were then resuspended in sample loading buffer and boiled for 5 min prior to sodium dodecyl sulfate polyacrylamide gel electrophoresis (SDS-PAGE). The biotinylated protein was used to blot the cell surface antigen expression and the total protein was used to blot the total antigen expression.

### Western blot analysis

Proteins were extracted with RIPA lysis buffer containing 1 mM PMSF (Beyotime, China). The protein samples were subjected to SDS-PAGE/immunoblotting analysis using anti-phospho-Smad1/5/8 antibody (1: 1000), anti-phospho-p38 antibody (1: 1000), anti-p38 antibody (1: 1000), anti-Runx2 antibody (1: 1000), anti-PPARγ antibody (1: 500), anti-C/EBPα antibody (1: 1000), anti-BMPR1A antibody (1: 250), anti-GAPDH antibody (1: 1000), or anti-β-actin antibody (1: 1000). The relative integrated density of each protein band was determined using an Odyssey infrared imaging system (LI-COR, NE, USA).

### Animal experiment

Thirty-six 10-week-old male Lewis rats were used in this experiment and were randomly allocated to three groups: a saline-injected group (*n* = 12), an lipopolysaccharide (LPS)-injected group (*n* = 12), and an IL-6-injected group (*n* = 12). The rhBMP-2/ACS implants were prepared as in previous studies^11,23^. Briefly, Type-I bovine ACS (Integra Life Sciences, USA), which are used as carriers for rhBMP-2, were tailored to dimensions of 10 × 10 × 5 mm. The sponges were then loaded with 100 μL of rhBMP-2 (1.5 mg/mL, which is equal to the clinically applied concentration of BMP-2) under sterile conditions. After the rats received the rhBMP-2/ACS implants, 100 μg of IL-6 in a volume of 200 μL or an equal volume of saline was subcutaneously injected into the implantation site. The rhBMP-2/ACS specimens were harvested at 4 and 8 weeks after implantation. The experimental procedures were approved by our institution’s Animal Research Committee.

### Histological and histomorphometry analyses

Specimens of mineral nodules were collected and fixed in 4% paraformaldehyde for 2 days. After rinsing within water, the specimens were decalcified in 20% EDTA for 30 days and embedded in paraffin. Serial 5-mm-thick sections were prepared at intervals of 80 μm and then stained with H&E. For quantitative analysis of the area of bone matrix and adipose tissue, H&E-stained sections were observed under a microscope at 200× magnification. The areas of bone matrix and adipose tissue were evaluated using Image-Pro Plus ver. 6.0 software (Media Cybernetics, MD, USA) in a blinded fashion.

### μCT scanning

The specimens were fixed in 4% paraformaldehyde for 2 days prior to μCT scanning (voxel size: 18 µm; SkyScan1176, Belgium). The resulting images were 2000 × 1048-pixel square images, and an aluminum-copper filter was employed to produce optimized images. Reconstructions and analyses were performed using NRecon reconstruction and CTAn 1.8 software, respectively. To measure newly formed bone, a circular area of a pre-defined size was selected as the region of interest (ROI) in the two-dimensional (2D) images. The pixel zone representing ossification in the defined ROI was then reconstructed in 3D by creating a volume of interest (VOI) in the lower and upper ranges of the threshold using grayscale units. After applying CTAn 1.8 to each reconstructed BMP file, the bone volume (BV) and bone mineral density (BMD) were obtained using a CT analyzer in direct 3D based on a surface-rendered volume model, according to the manufacturer’s instructions. In addition, the total bone mineral content (BMC) was calculated by multiplying the BV by the BMD.

### Statistical analyses

The data are expressed as the mean ± s.d. of at least three independent experiments. Student’s *t-*test was used to determine significance between two groups. *P*-values < 0.05 were considered statistically significant. IBM SPSS statistics ver. 20 was used for the statistical analyses.

## Results

### IL-6 injection enhances new bone regeneration and induces excessive adipose tissue formation

**Fig. 1:**
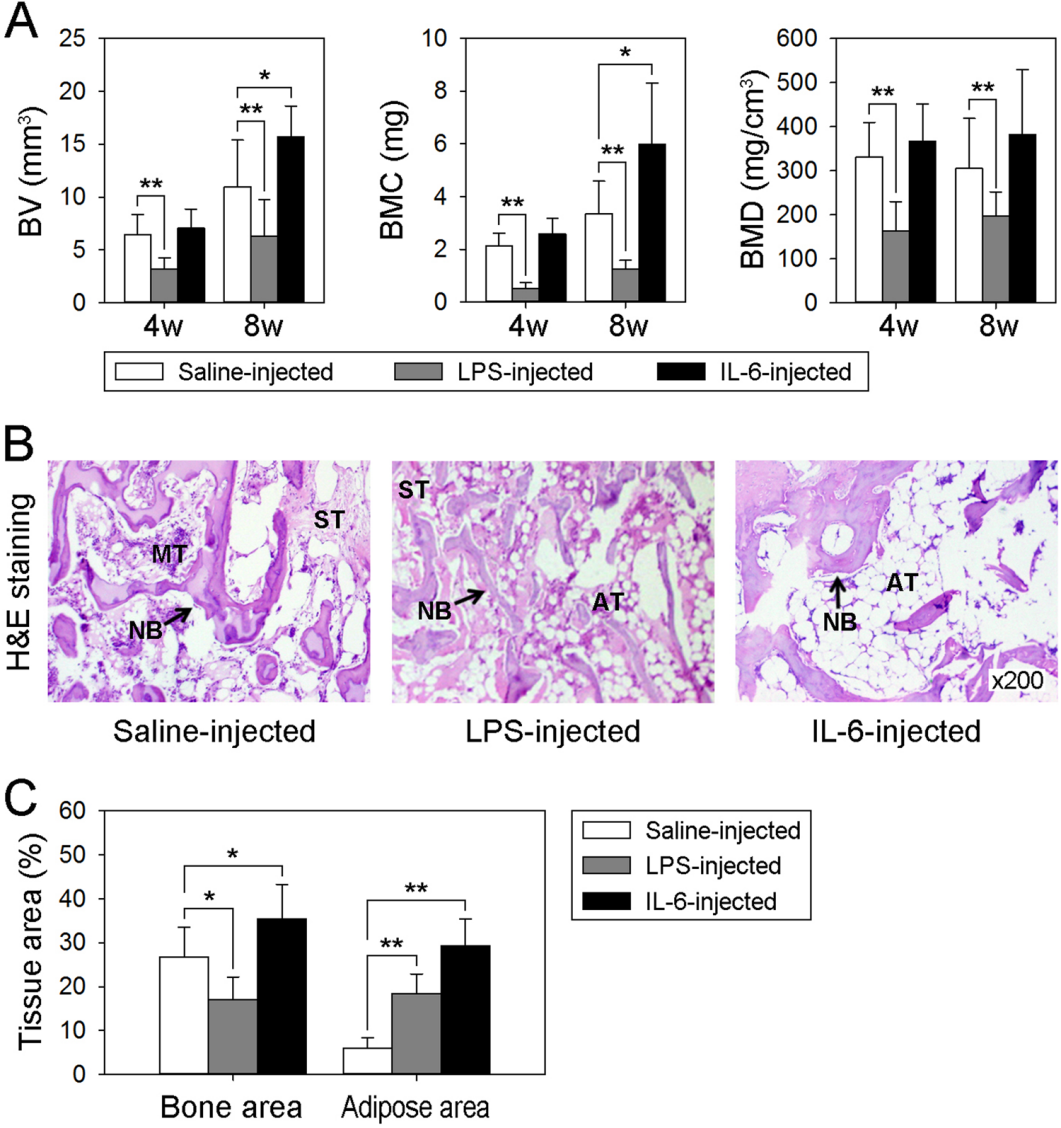
IL-6 injection enhances rhBMP-2/ACS-induced bone regeneration and induces excessive adipose tissue formation in a rat model **a** The BV, BMD, and BMC of the rhBMP-2/ACS implants harvested at 4 and 8 weeks, as measured by μCT. **b** Representative H&E-stained images of rhBMP-2/ACS implants harvested at 8 weeks. **c** Histomorphometric analysis of the area of bone matrix and adipose tissue. *P < 0.05, **P < 0.01, compared with the saline-injected groups. (NB new bone, ST soft tissue, MT mesenchymal tissue, AT adipose tissue)

To explore the effects of an elevated IL-6 level on rhBMP-2/ACS-induced osteogenesis and adipogenesis in vivo, we established an ectopic bone formation model in rats via rhBMP-2/ACS implantation, as described in previous studies^11,23^. As shown in Fig. 1a, LPS injection, performed to mimic the exaggerated inflammatory environment under clinical conditions, significantly decreased the BV, BMC, and especially BMD of the rhBMP-2/ACS-regenerated mineral nodules at 4 and 8 weeks post-implantation, indicating that rhBMP-2/ACS-induced bone regeneration in vivo but the exaggerated inflammatory environment impaired bone quality. In contrast, IL-6 injection, which was conducted to artificially increase the level of IL-6 surrounding the rhBMP-2/ACS implant, obviously increased the BV and BMC of the mineral nodules but did not affect BMD at 8 weeks post-surgery, demonstrating an elevated IL-6 level enhanced rhBMP-2/ACS-induced bone regeneration. Histologically, both newly formed bone matrix and adipose tissue were observed in the H&E-stained images from all three groups of mineral nodules (Fig. 1b). However, histomorphometric measurements revealed differences in the bone area and fat area in histological sections. Compared with the saline-injected group, LPS injection resulted in a decrease in the bone matrix area but an increase in the adipose tissue area, and IL-6 injection led to increases in both the bone matrix and adipose tissue areas (Fig. 1c).

### The presence of IL-6/sIL-6R strongly enhances rhBMP-2-induced osteogenic and adipogenic commitment and differentiation

The biological effects of rhBMP-2 on adipo-osteogenic differentiation of hBMSCs were investigated before presence of IL-6 and sIL-6R were present. The osteoinduction and adipoinduction protocol of hBMSCs protocol is shown in Supplementary Fig. 1A-B. As illustrated in Supplementary Fig. 1C-E, the hBMSCs cultured in ODM alone showed high ALP activity and calcium deposition. However, pretreatment with rhBMP-2 during proliferation significantly enhanced the ODM-induced osteogenic differentiation of hBMSCs, and the peak osteoinductive concentration of rhBMP-2 was 200 ng/mL. Quantitative analysis of ALP activity (Supplementary Fig. 1D) and ARS staining (Supplementary Fig. 1F) further showed the osteoinductive capacity of rhBMP-2. Most interestingly, pretreatment of hBMSCs with rhBMP-2 during proliferation also showed a positive and dose-dependent influence on the ADM-induced adipogenesis of hBMSCs (Supplementary Fig. 1G-H). The positive role of rhBMP-2 in the adipo-osteogenic differentiation of hBMSCs was further confirmed via genetic analysis after 6 days in ODM or ADM by examining the expression patterns of the osteogenic differentiation markers osteopontin (OPN) and osteocalcin (OCN) and the adipogenic differentiation markers adipocyte fatty acid-binding protein-2 (aP2) and C/EBPβ. Consistent with the results of cell staining, significant induction of these genes relative to undifferentiated cells and ODM- or ADM-treated cells was observed after rhBMP-2 treatment was observed (Supplementary Fig. 2A-D). Based on these data, we chose 200 ng/mL as the final concentration of rhBMP-2 for the following adipo-osteogenic differentiation experiments. These data again confirmed that again rhBMP-2 optimizes both the osteogenic and adipogenic differentiation of hBMSCs.

**Fig. 2:**
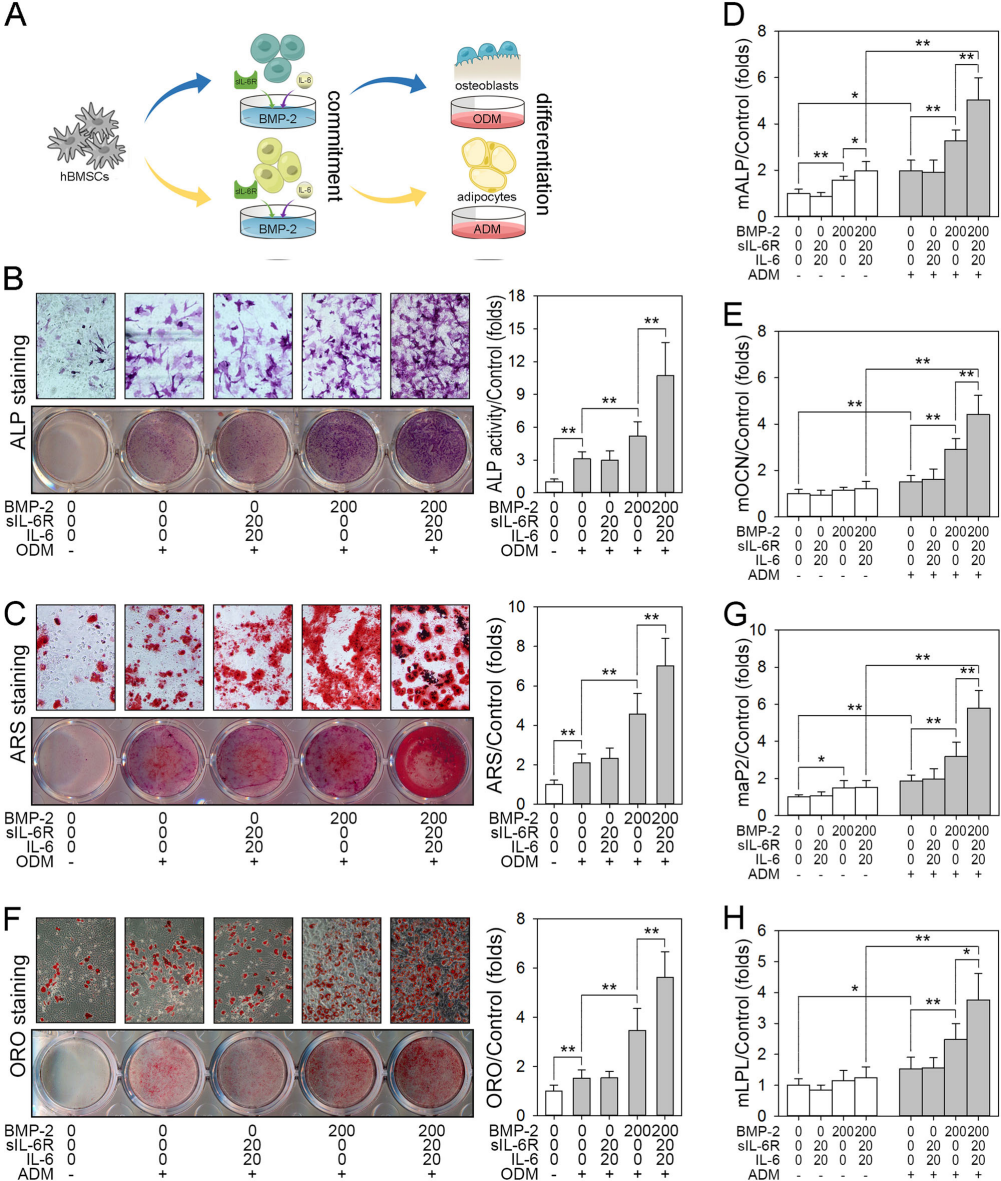
The presence of IL-6/sIL-6R strongly enhances rhBMP-2-induced osteogenic and adipogenic differentiation in vitro **a** hBMSCs were plated at a low density and treated with rhBMP-2 in the presence or absence of IL-6/sIL-6R for 3 days. Then, postconfluent cells were cultured with ODM or ADM. **b** An ALP staining assay was performed to measure ALP activity on day 7, with the relative quantification of ALP activity was measured on day 3, normalized against untreated cells. **c** ARS staining was performed to measure calcium deposition on day 21, with the relatively quantified ARS staining, normalized against the untreated cells. **d**–**e** Confluent hBMSCs were treated with ODM for 48 h. Real-time PCR analysis of ALP (**d**) and OCN (**e**). **f** ORO staining was performed to measure lipid accumulation on day 18, and the with relatively quantified ORO level was normalized against untreated cells. **g**–**h** Confluent hBMSCs were treated with ODM for 48 h. Real-time PCR analysis of aP2 (**g**) and LPL (**h**) was performed. *P < 0.05, **P < 0.01, compared with untreated cells or the indicated groups

Our previous study demonstrated that IL-6 and sIL-6R synergistically enhance the rhBMP-2-induced osteogenic differentiation of hBMSCs^23^. To further investigate the convergence of IL-6/sIL-6R and rhBMP-2 on the promotion of osteogenesis and adipogenesis, hBMSCs were pretreated with rhBMP-2 in the presence or absence of IL-6/sIL-6R and then subjected to osteogenic or adipogenic differentiation (Fig. 2a). As shown in Fig. 2b, c, pretreatment with rhBMP-2 alone increased ODM-induced ALP expression and calcium deposition. Interestingly, this enhanced osteogenesis was further promoted by the addition of IL-6/sIL-6R, with a 3.2-fold increase in ALP activity and a 3.6-fold increase in ARS staining being observed. The quantitative analysis of the gene expression was consistent with the observed staining, and the combination of rhBMP-2 and IL-6/sIL-6R resulted in an even greater increase in ALP and OCN mRNA expression (Fig. 2d, e). Adipogenic differentiation was also investigated after pretreatment with rhBMP-2 either alone or in combination with IL-6/sIL-6R. Similar with the osteogenic differentiation environment, the rhBMP-2-induced adipogenic differentiation was further strengthened by the presence of IL-6/sIL-6R (Fig. 2f–h). In summary, these data suggest that rhBMP-2 combined with IL-6/sIL-6R optimizes the osteogenic and adipogenic differentiation of hBMSCs.

**Fig. 3:**
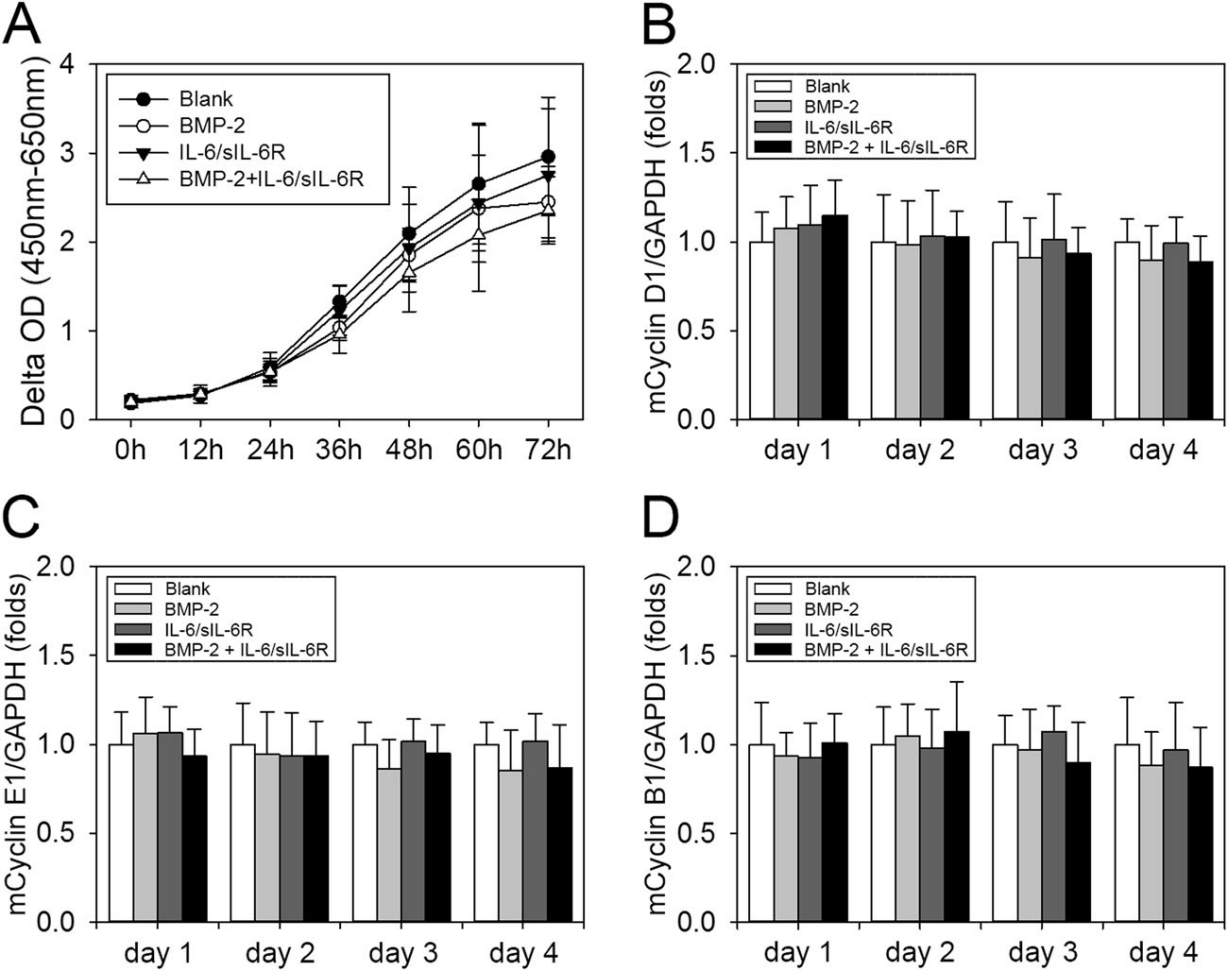
rhBMP-2, IL-6/sIL-6R, and the combination of rhBMP-2 and IL-6/sIL-6R have no effect on hBMSC proliferation **a** hBMSCs were plated at a low density and treated with rhBMP-2 in the presence or absence of IL-6/sIL-6R for 3 days. Then, CCK-8 assays were performed to analyze cell growth. **b**–**d** hBMSCs were treated as previously described, and then real-time PCR analysis of Cyclin D1 (**b**), Cyclin E1 (**c**), and Cyclin B1 (**d**) was performed

To determine whether the enhanced osteogenic and adipogenic commitment of hBMSCs were due to the synergistic effect of rhBMP-2 and IL-6/sIL-6R or cell proliferation, cell growth was assessed via CCK-8 assays after treatment with rhBMP-2 alone, IL-6/sIL-6R, or a combination of rhBMP-2 and IL-6/sIL-6R. As shown in Fig. 3a, treatment with rhBMP-2, IL-6/sIL-6R, or a combination of rhBMP-2 and IL-6/sIL-6R did not seem to affect cell growth up to 72 h, suggesting that none of these treatments affect cell proliferation in our cell model. To obtain further support for this hypothesis, the mRNA levels of cyclins D1, E1, and B1 were quantified via quantitative RT-PCR. The RT-PCR results were consistent with those of the CCK-8 assay, demonstrating that treatment with rhBMP-2, IL-6/sIL-6R, or a combination of rhBMP-2 and IL-6/sIL-6R had no influence on the mRNA expression of the cyclins D1, E1, and B1 during proliferation (Fig. 3b–d).

### IL-6/sIL-6R potentiates the rhBMP-2-induced osteogenic and adipogenic commitment of hBMSCs by promoting the cell surface translocation of BMPR1A

**Fig. 4:**
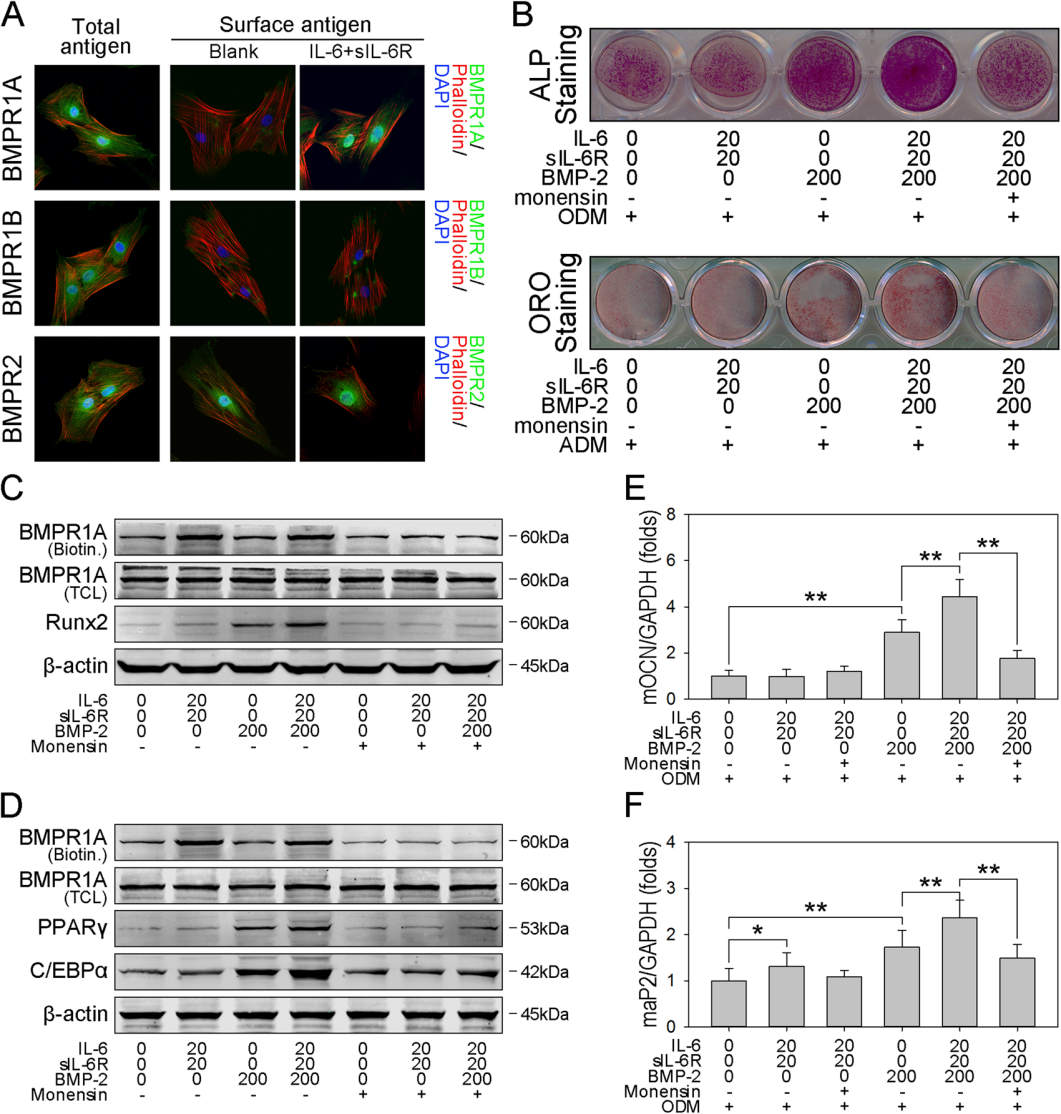
IL-6/sIL-6R-induced cell surface translocation of BMPR1A enhances rhBMP-2-induced osteogenic and adipogenic differentiation **a** hBMSCs were plated at a low density and treated with or without IL-6/sIL-6R for 48 h. Then, cells were fixed and treated with or without 1% Triton X-100 for 30 min, followed by immunofluorescence analysis to detect the total or cell surface antigen levels of BMPR1A, BMPR1B, and BMPR2. Original magnification:200×. **b** hBMSCs were plated at a low density and treated with rhBMP-2 in the presence or absence of IL-6/sIL-6R and monensin for 3 days. Then, postconfluent cells were cultured with ODM or ADM. An ALP staining assay was performed in the ODM-cultured cells on day 7 to measure ALP activity, and ORO staining was performed in the ADM-cultured cells on day 18 to measure lipid accumulation. **c** Postconfluent hBMSCs were treated with rhBMP-2 in the presence or absence of IL-6/sIL-6R and monensin for 3 days, and immunoblotting analysis of biotinylated BMPR1A, total BMPR1A, and Runx2 was performed and normalized against β-actin. **d** Postconfluent hBMSCs were treated as previously described, and immunoblotting analysis of biotinylated BMPR1A, total BMPR1A, PPARγ, and C/EBPα was performed and normalized against β-actin. **e**–**f** hBMSCs were plated at a low density and treated with rhBMP-2 in the presence or absence of IL-6/sIL-6R and monensin for 3 days. Then, postconfluent cells were cultured with ODM or ADM, and real-time PCR analysis of OCN (**e**) and aP2 (**f**) was performed. *P < 0.05, **P < 0.01, compared with untreated cells or the indicated groups

The BMP-2 signaling cascade is transduced via three specific transmembrane serine/threonine kinase receptors, BMPR1A, BMPR1B, and BMPR2. As previously reported, IL-6/sIL-6R can promote the cell surface localization of BMPR1A, but does not affect BMPR1B and BMPR2^23^. To build on these findings, immunofluorescence analyses were performed to investigate the cell membrane distribution and expression level of BMPRs. As shown in Fig. 4a, BMPR1A and BMPR1B were mainly located in the cell cytoplasm. In contrast, BMPR2 was predominantly located at the cell surface. Strikingly, IL-6/sIL-6R treatment significantly increased the cell surface localization of BMPR1A, but not that of BMPR1B or BMPR2 (Fig. 4a).

To confirm that the observed cell surface translocation of BMPR1A was associated with rhBMP-2-induced osteogenesis and adipogenesis, the intracellular protein transport inhibitor monensin was added to the cell medium during proliferation to block IL-6/sIL-6R-induced BMPR1A cell surface translocation. After osteogenesis and adipogenesis induction, the presence of IL-6/sIL-6R potentiated rhBMP-2-induced ALP activity and lipid droplet formation. However, monensin reversed the effect of IL-6/sIL-6R to basal levels (Fig. 4b), indicating that IL-6/sIL-6R potentiates rhBMP-2-induced osteogenesis and adipogenesis through regulating BMPR1A cell surface translocation. To confirm this hypothesis, the expression levels of the osteogenic commitment marker Runx2 and the adipogenic commitment markers PPARγ and C/EBPα were analyzed in hBMSCs pretreated with a combination of rhBMP-2 and IL-6/sIL-6R. As shown in the biotinylation band (Fig. 4c, d), the presence of IL-6/sIL-6R caused a significant synergistic effect on the cell surface translocation of BMPR1A, revealed by accumulating biotinylated BMPR1A (lanes 2 and 4). As expected, the addition of monensin decreased the protein level of biotinylated BMPR1A (lanes 5–7) and markedly reversed the elevation of Runx2, PPARγ, and C/EBPα levels to basal levels (lane 7). The quantitative analysis of osteogenesis- and adipogenesis-related gene expression was consistent with the tendency observed in the western blot analysis, as shown by significant downregulation of the mRNA expression levels of OCN and aP2 in monensin-supplemented cells (Fig. 4e–f).

### BMP/Smad signaling is required for BMPR1A-mediated osteogenic commitment and differentiation

**Fig. 5:**
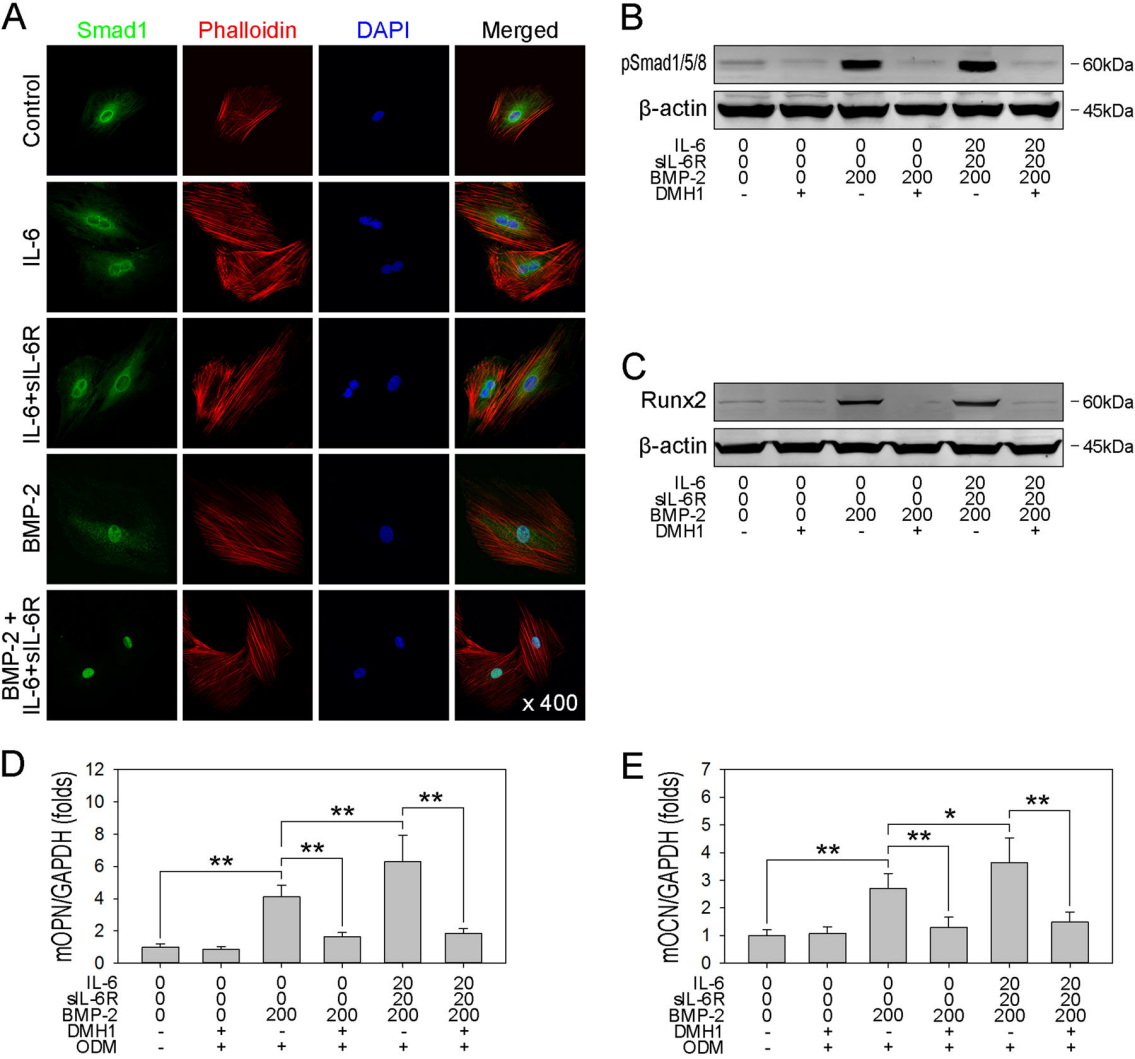
BMP/Smad signaling is required for rhBMP-2-induced osteogenic differentiation **a** hBMSCs were plated at a low density and treated with rhBMP-2, IL-6, IL-6/sIL-6R or a combination of IL-6/sIL-6R and rhBMP-2 for 30 min, and then immunofluorescence analysis of Smad1 was performed. Original magnification: 200×. **b** Postconfluent hBMSCs were treated with rhBMP-2 in the presence or absence of IL-6/sIL-6R and DMH1 for 60 min, and then immunoblotting analysis of pSmad1/5/8 was performed and normalized against β-actin. **c** Postconfluence hBMSCs were treated with rhBMP-2 in the presence or absence of IL-6/sIL-6R and DMH1 for 3 days, and then immunoblotting analysis of Runx2 was performed and normalized against β-actin. **d**–**e** hBMSCs were plated at a low density and treated with rhBMP-2 in the presence or absence of IL-6/sIL-6R and DMH1 for 3 days. Then, the postconfluent cells were cultured with ODM, and real-time PCR analysis of OPN (**d**) and OCN (**e**) was performed. *P < 0.05, **P < 0.01, compared with untreated cells or the indicated groups

To evaluate the role of IL-6/sIL-6R in the signal transduction induced by BMPR1A translocation, proliferating hBMSCs were treated with rhBMP-2 either alone or together with IL-6/sIL-6R. As shown in Fig. 5a, Smad1/5/8, a known downstream target of BMP-2, was rapidly phosphorylated and subsequently transported to the nucleus after rhBMP-2 treatment. To confirm that the augmented osteogenic differentiation of hBMSCs results in signaling via the BMPR1A-mediated BMP/Smad pathway, DMH1, a highly selective BMP receptor inhibitor^32^, was used to block the BMP/Smad pathway. As expected, treatment with DMH1 effectively caused phosphorylated Smad1/5/8 to reach a basal level in both the rhBMP-2-treated cells and the rhBMP-2/IL-6/sIL-6R-treated cells (Fig. 5b), indicating complete blockade of the BMP/Smad pathway. Then, the commitment of hBMSCs was assessed via immunoblotting. As shown in Fig. 5c, the blockade of BMP/Smad signaling offset the positive effect and reversed the elevation of Runx2 to basal levels. Furthermore, the addition of DMH1 significantly decreased the mRNA expression of OPN and OCN to basal levels (Fig. 5d, e). These findings indicate that the cell surface translocation of BMPR1A amplifies the BMP/Smad pathway, which is responsible for the enhanced osteogenesis of hBMSCs.

### rhBMP-2-induced adipogenic commitment is independent on BMP/Smad signaling

**Fig. 6:**
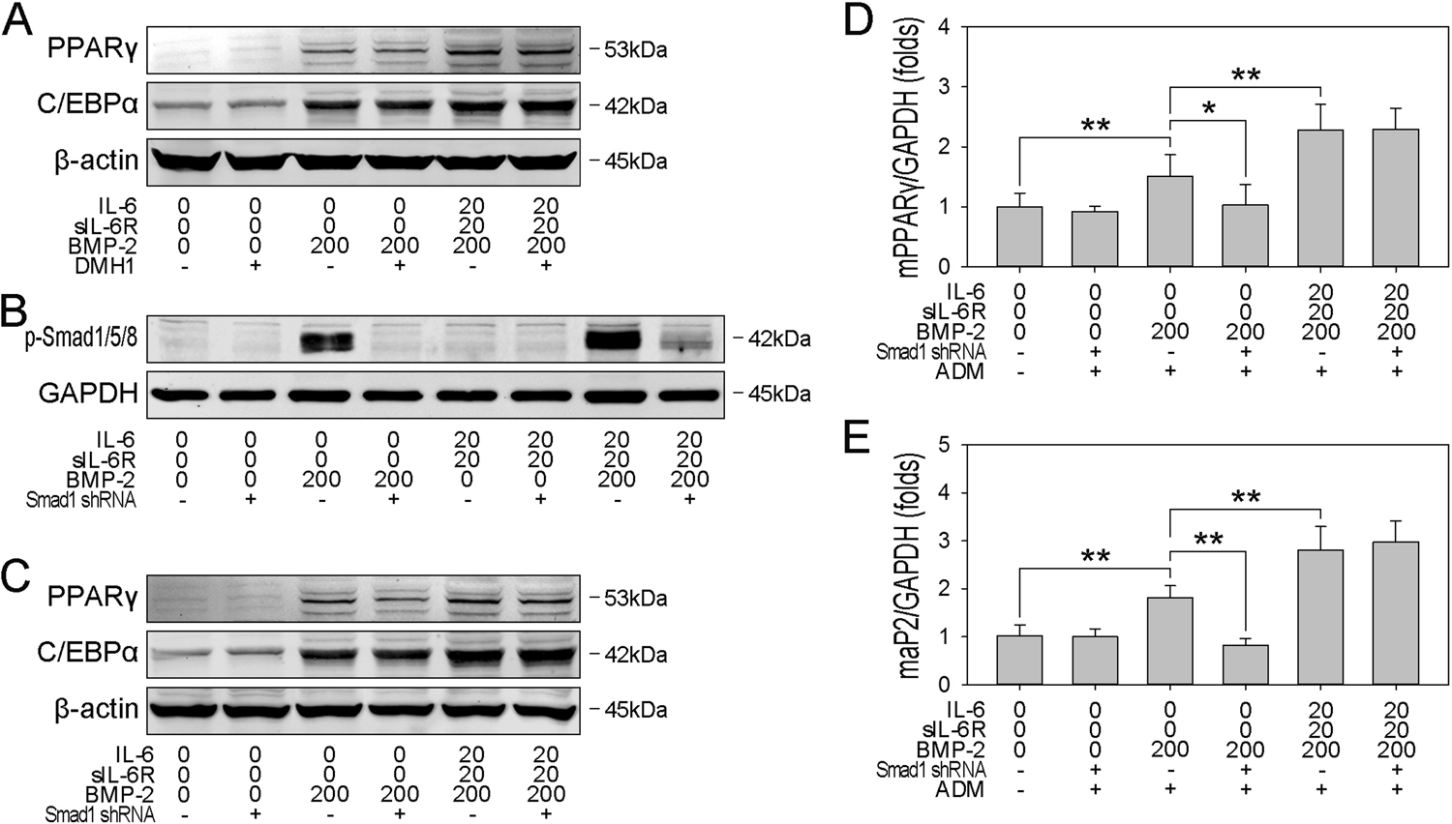
IL-6/sIL-6R enhances rhBMP-2-induced adipogenic differentiation independent of BMP/Smad signaling **a** Postconfluent hBMSCs were treated with rhBMP-2 in the presence or absence of IL-6/sIL-6R and DMH1 for 3 days, and then immunoblotting analysis of PPARγ and C/EBPα was performed and normalized against β-actin. **b** hBMSCs were cotransfected with plasmids containing Smad1 for 24 h, and then immunoblotting analysis of pSmad1/5/8 in the cell lysates was performed after a 60-min treatment (normalized against GAPDH, lower plane). **c** hBMSCs were transfected with plasmids expressing Smad1 shRNA or empty vectors. Then, the cells were treated with rhBMP-2 in the presence or absence of IL-6/sIL-6R for 3 days, and then immunoblotting analysis of PPARγ and C/EBPα was performed and normalized against β-actin. **d**–**e** hBMSCs were transfected with plasmids containing Smad1 shRNA or empty vectors. Then, the cells were treated with rhBMP-2 in the presence or absence of IL-6/sIL-6R for 3 days, and then the postconfluent cells were cultured with ADM, and real-time PCR analysis of PPARγ (**d**) and aP2 (**e**) was performed. *P < 0.05, **P < 0.01, compared with untreated cells or the indicated groups

To ascertain whether the observed optimized adipogenesis in hBMSCs results from amplification of the BMP/Smad pathway, adipogenesis was evaluated by examining the expression of PPARγ, C/EBPα, and aP2 at the protein and mRNA levels. DMH1 was also used to block BMP/Smad signaling in rhBMP-2-treated hBMSCs, which were subsequently submitted for adipogenic differentiation. In contrast to osteogenesis, DMH1 had no obvious inhibitory effect on rhBMP-2-induced adipogenesis, characterized by PPARγ and C/EBPα expression (Fig. 6a). However, given that DMH1 blocks the activation of the BMP type-I receptor ALK2, to eliminate the underlying effect on other BMP-2-activated signaling pathways, Smad1 shRNA was transfected to hBMSCs to silence the expression of Smad1 and block the activation of BMP/Smad signaling pathway. As expected, Smad1 shRNA transfection attenuated the rhBMP-2-induced phosphorylation level of Smad1/5/8 (Fig. 6b). More importantly, transfection of Smad1 shRNA also significantly decreased the rhBMP-2-induced protein expression of PPARγ and C/EBPα in protein level (Fig. 6c), and mRNA expression of PPARγ and aP2 (Fig. 6d, e). In summary, these observations suggest that the BMP/Smad pathway is not required for BMPR1A-mediated commitment and differentiation.

### Cell surface translocation of BMPR1A enhances adipogenic commitment through p38 MAPK

**Fig. 7:**
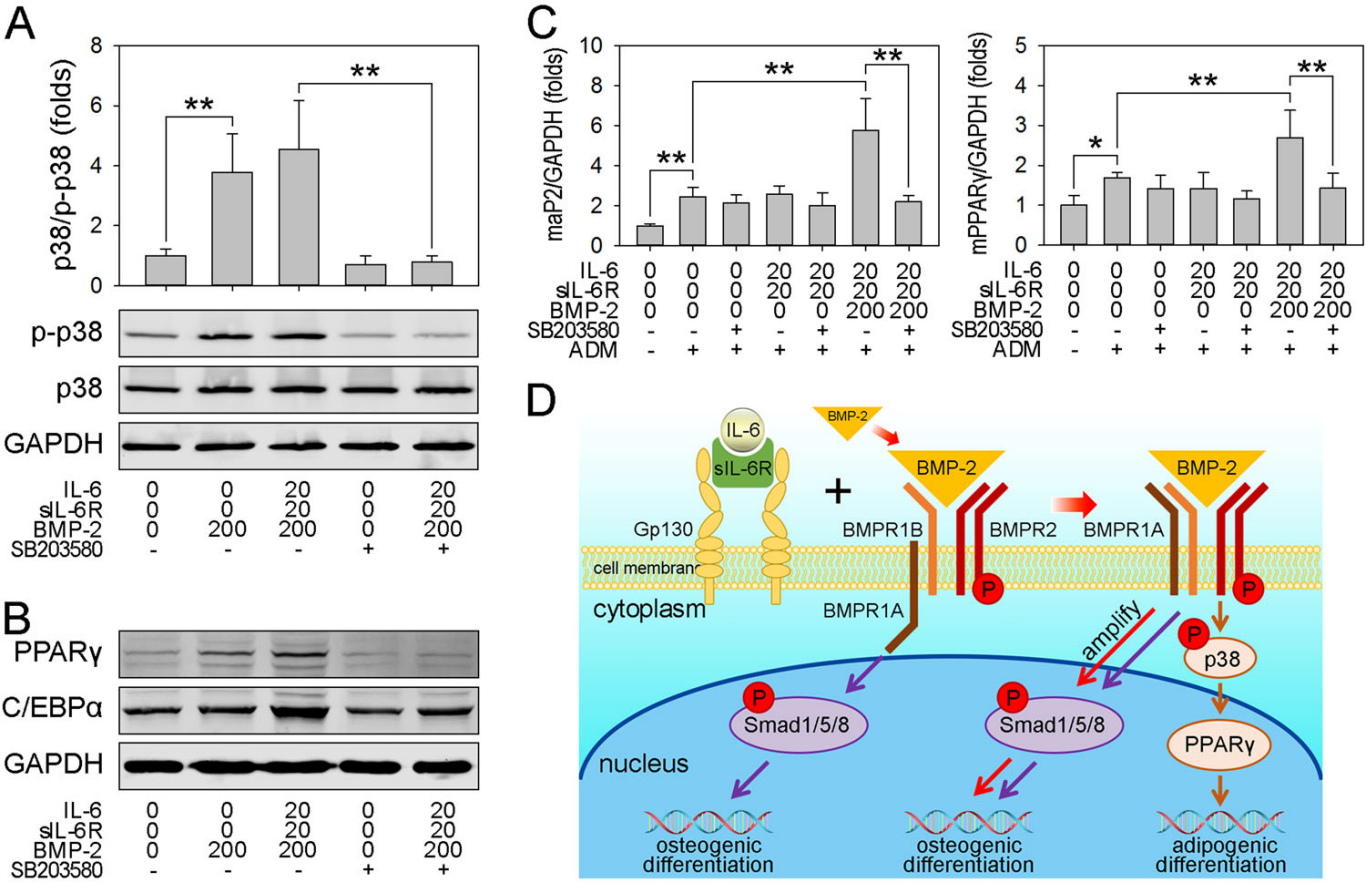
The cell surface translocation of BMPR1A enhances adipogenic differentiation through p38 MAPK **a** Postconfluent hBMSCs were treated with rhBMP-2 in the presence or absence of IL-6/sIL-6R and SB203580 for 30 min, and then immunoblotting analysis of p38 and p-p38 was performed (lower plane), and the relative expression of phosphorylated p38 was quantified (upper plane), normalized against GAPDH. **b** Postconfluent hBMSCs were treated with rhBMP-2 in the presence or absence of IL-6/sIL-6R and SB203580 for 3 days, and then immunoblotting analysis of PPARγ and C/EBPα was performed and normalized against GAPDH. **c** hBMSCs were plated at a low density and treated with rhBMP-2 in the presence or absence of IL-6/sIL-6R and SB203580 for 3 days. The postconfluent cells were cultured with ADM, and then real-time PCR analysis of aP2 and PPARγ was performed. *P < 0.05, **P < 0.01, compared with untreated cells or the indicated groups. **d** IL-6 potentiates BMP-2-induced osteogenesis by promoting the cell surface localization of BMPR1A, which subsequently amplifies BMP/Smad signaling. However, IL-6 enhances BMP-2-induced adipogenesis through BMPR1A-mediated p38 MAPK

We previously reported that BMP-2 activates both the BMP/Smad and p38 MAPK pathways^22^. In this study, we observed that rhBMP-2 alone rapidly provoked the phosphorylation of p38, and the presence of IL-6/sIL-6R slightly enhanced this effect. However, treatment with SB203580, a p38 MAPK inhibitor, significantly decreased the phosphorylation of p38 below basal levels (Fig. 7a). To assess the role of p38 MAPK in BMPR1A-enhanced adipocyte commitment, cell lysis was performed to assess the protein levels of PPARγ and C/EBPα. However, SB203580 not only decreased the phosphorylation of p38 but also significantly impaired the adipogenic commitment of hBMSCs, as demonstrated by decreases in the protein levels of PPARγ and C/EBPα (Fig. 7b) and mRNA levels of PPARγ and aP2 (Fig. 7c). These findings indicate that BMPR1A cell surface translocation also activates p38 MAPK, which is responsible for the enhanced adipogenesis of hBMSCs.

## Discussion

The working hypothesis in this study was that the commitment to new bone and adipose tissue formation at sites that receive rhBMP-2 treatment results from a high rhBMP-2 concentration and an elevated IL-6 level in the exaggerated inflammatory environment. To investigate this hypothesis, a cell model and an animal model were used to evaluate the role of IL-6 in rhBMP-2-mediated osteogenesis and adipogenesis. Our results revealed that IL-6 potentiates rhBMP-2-induced osteogenesis and adipogenesis both in vivo and in vitro. Furthermore, we demonstrated that IL-6/sIL-6R treatment promotes the cell surface translocation of BMPR1A and subsequently augments rhBMP-2-induced osteogenic commitment through amplifying BMP/Smad signaling and rhBMP-2-induced adipogenic commitment via p38 MAPK signaling (Fig. 7d). These findings provide new insights into why high doses of rhBMP-2 cause adverse events in humans and may provide an alternative approach for improving the osteoinductive efficiency of rhBMP-2 in clinical applications.

**Fig. 8:**
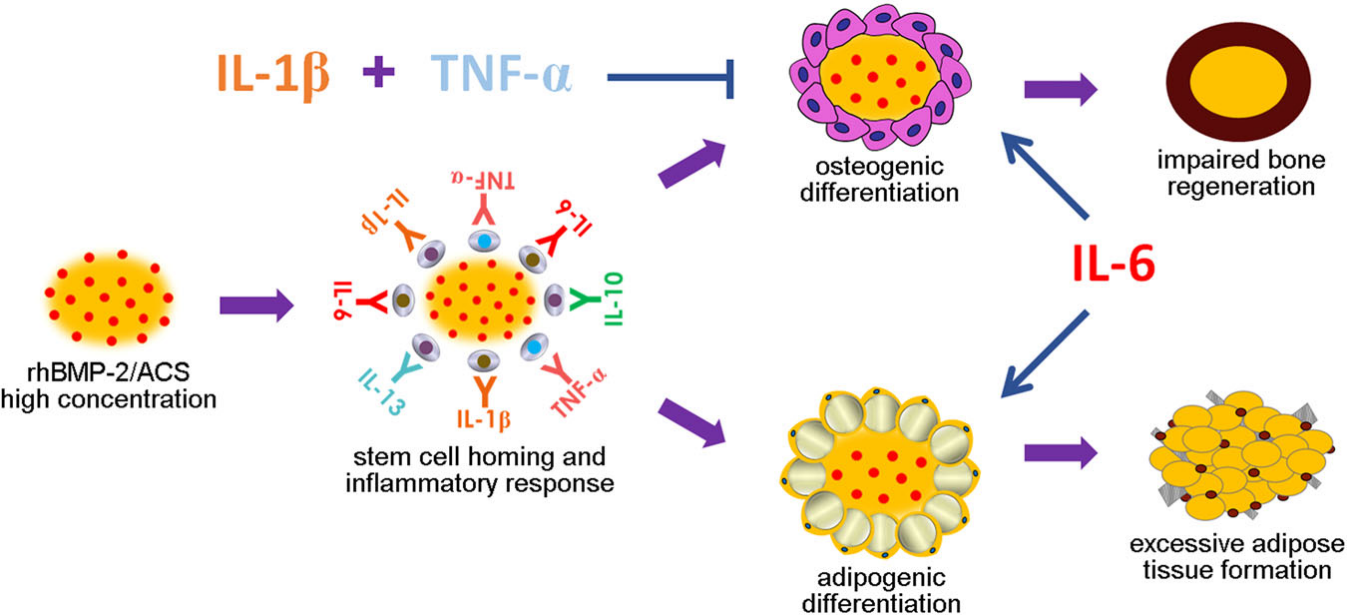
A high dose of rhBMP-2 provokes an exaggerated inflammatory environment, which subsequently results in impaired bone quality and excessive adipose tissue formation The application of a high dose of rhBMP-2 in clinical conditions results in an exaggerated inflammatory environment, characterized by the secretion of inflammatory cytokines and infiltration of inflammatory cells. The exaggerated inflammatory environment results in the formation of crystal-like bone voids and impairs the quality of rhBMP-2-induced bony tissue through secretion of inflammatory cytokines, such as TNF-α and IL-1β. IL-6 concomitantly promotes rhBMP-2-induced adipogenesis and excessive adipose tissue formation in bone voids

Although rhBMP-2 induces promising bone formation in clinical settings, deteriorated bone quality and enhanced adipose tissue formation are also observed at sites receiving high-dose rhBMP-2 treatment^5,16,17^. This phenomenon was corroborated by our in vitro results and published data indicated that rhBMP-2 can induce osteogenesis in addition to, or instead of, adipogenesis^19,20,33^. These findings indicate that the osteogenic and adipogenic potential of hBMSCs may exhibit a reciprocal relationship. More importantly, our data verified that IL-6 and sIL-6R synergistically enhanced the rhBMP-2-induced osteogenic and adipogenic differentiation of hBMSCs. Similar results have been reported for BMP-7, an osteoinductor with effects similar to BMP-2, with IL-6/sIL-6R acting synergistically with BMP-7 to stimulate rat osteoblastic cell differentiation^34^. Based on our previous work and these results, the occurrence of new bone formation, deteriorated bone quality, and adipose tissue formation in clinical conditions can be interpreted at the cellular level. The application of rhBMP-2 induces new bone formation at implantation sites. However, the high rhBMP-2 concentration results in an exaggerated inflammatory response, subsequently leading to the formation of crystal-like bone voids and impairing the quality of rhBMP-2-induced bony tissue through the secretion of inflammatory cytokines, such as TNF-α and IL-1β^11,14^. Additionally, IL-6, which is significantly elevated in the exaggerated inflammatory environment^11,21^, concomitantly promotes rhBMP-2-induced adipogenesis, followed by excessive adipose tissue formation in bone voids (Fig. 8)^15^. In the current study, we also observed that IL-6/sIL-6R enhanced rhBMP-2-induced osteogenesis, which seems to conflict with the widely accepted idea that the exaggerated inflammatory environment plays a negative role in bone regeneration. The most logical explanation is that the inhibitory effect of the inflammatory environment on rhBMP-2/ACS-induced bone regeneration is due to the combined effects of different inflammatory cytokines. However, the effect of each inflammatory cytokine on rhBMP-2/ACS -induced bone regeneration may differ.

BMSCs are multipotent cells capable of differentiating into mature cells of several mesenchymal tissue types, such as fat, cartilage, and bone. As common progenitor cells of adipocytes and osteoblasts, BMSCs are delicately balanced for differentiation commitment. The developmental pathway from BMSCs to mature cells involves in two distinct stages: lineage commitment (from BMSCs to lineage-specific progenitors) and terminal differentiation (from progenitors to specific cell types). Therefore, we established a cell culture model for this study in which hBMSCs were first treated with rhBMP-2 in the presence or absence of IL-6/sIL-6R during proliferation, leading to commitment to osteogenic or adipogenic progenitors (preosteoblasts or preadipocytes, respectively). Three days after reaching confluence, the cell culture medium was changed to standard ODM or ADM for terminal differentiation (Fig. 2a). Similar commitment and differentiation models have been used to study the BMP-2/4-induced adipo-osteogenic commitment and differentiation of MSCs^35,36^.

Acting as one of the three transmembrane receptors of BMPs, BMPR1A plays a vital role in activating BMP-2-mediated signaling cascades and embryonic development, including osteogenesis, chondrogenesis, and adipogenesis^37–39^. Cell surface translocation of BMPRs modulates the responsiveness of target cells to growth factors. Our previous work showed that IL-6/sIL-6R promotes the translocation of BMPR1A from the cell cytoplasm to the cell surface, which subsequently amplifies the BMP-2-activated BMP/Smad signaling pathway and ultimately leads to enhanced osteogenic differentiation of hBMSCs^23^. In the present study, we confirmed the existence of this positive feedback between IL-6/sIL-6R treatment and rhBMP-2-induced osteogenic lineage commitment. Furthermore, our data demonstrated that the translocation of BMPR1A augments PPARγ expression and, subsequently, adipogenic lineage commitment. This finding is consistent with a report in which phosphorylation of BMPR1A through mutation of the highly conserved, ubiquitously expressed enzyme Casein kinase II was shown to lead to both adipogenesis and osteogenesis, even without BMP-2 stimulation^40^. The positive role of BMPR1A in adipogenesis has been further validated, as increased BMPR1A expression during adipogenesis is observed in overweight and obese individuals^41,42^. Additionally, the enrichment of adipose-derived stromal cells for BMPR1A facilitates enhanced adipogenesis^43^, and BMP-2/4 stimulation can induce the adipogenic commitment of C3H10T1/2 stem cells through BMPR1A^35^. In contrast to these findings, in our study, the enhancement of adipogenesis resulted from the cell surface translocation of BMPR1A and not increased BMPR1A expression.

BMPR1A-mediated downstream pathways are critical for both osteogenesis and adipogenesis. BMP-2 first initiates the phosphorylation of BMPR1A at the cell membrane and subsequently activates the canonical BMP/Smad pathway^39^ and a non-canonical p38 MAPK pathway^44^. Both pathways modulate BMP-2-induced adipo-osteogenic differentiation of MSCs^35^. In our cell model, the presence of IL-6/sIL-6R amplified the BMP-2-activated BMP/Smad and p38 MAPK pathways. However, the two amplified pathways exert different influences on the commitment and differentiation of hBMSCs. The amplified BMP/Smad pathway led to enhanced Runx2 expression and subsequent osteogenesis in hBMSCs but did not affect adipogenesis. However, the amplified p38 MAPK pathway resulted in the upregulation of PPARγ, along with adipogenesis. Our results were similar, but different, to those of several previous studies showing that the BMP/Smad pathway is concomitantly activated with the p38 MAPK pathway and both pathways are responsible for BMP-2/4-induced osteogenesis and adipogenesis^35,45^. In our culture system, to mimic the clinical setting after application of high-dose rhBMP-2, the concentration of rhBMP-2 was much higher than that used for the induction of adipogenesis. Therefore, the cell signal pathways activated by different rhBMP-2 concentrations may be different in the same cell model. In other words, a low-dose of rhBMP-2 mainly activates the BMP/Smad pathway and then leads to adipogenic commitment. However, a high dose of rhBMP-2 concomitantly activates both the BMP/Smad and p38 MAPK pathways but plays distinct roles in controlling the osteogenic and adipogenic commitment of hBMSCs. A previous report partly supported our hypotheses in which BMP-2 treatment regulates the transcriptional activity of PPARγ though p38 MAPK signaling, but not BMP signaling, in C3H10T1/2 cells^46^.

## Conclusion

In the present study, we determined that IL-6 potentiates rhBMP-2-induced osteogenesis and adipogenesis through the BMPR1A-mediated BMP/Smad pathway and the p38 MAPK pathway. These findings provide a potential interpretation for the concomitant occurrence of new bone and adipose tissue formation after clinical application of rhBMP-2/ACS, which may result from the elevation of IL-6 in the exaggerated inflammatory environment. Our findings provide molecular insights into the role of the inflammatory environment linking rhBMP-2-induced osteogenesis and adipogenesis. However, this study has several limitations. Related issues, such as the need for further details on the signaling crosstalk among BMPR1A, the BMP/Smad pathway and the p38 MAPK pathway, will require further investigation.

## Electronic supplementary material

Supplementary Figure 1

Supplementary Figure 2

Supplementary information
