# Supplementary figures and images for: IL-6 potentiates BMP-2-induced osteogenesis and adipogenesis via two different BMPR1A-mediated pathways
Source: Cell Death Dis. 2018 Feb 2;9(2):144. doi: 10.1038/s41419-017-0126-0 (PMC5833364; doi:10.1038/s41419-017-0126-0)

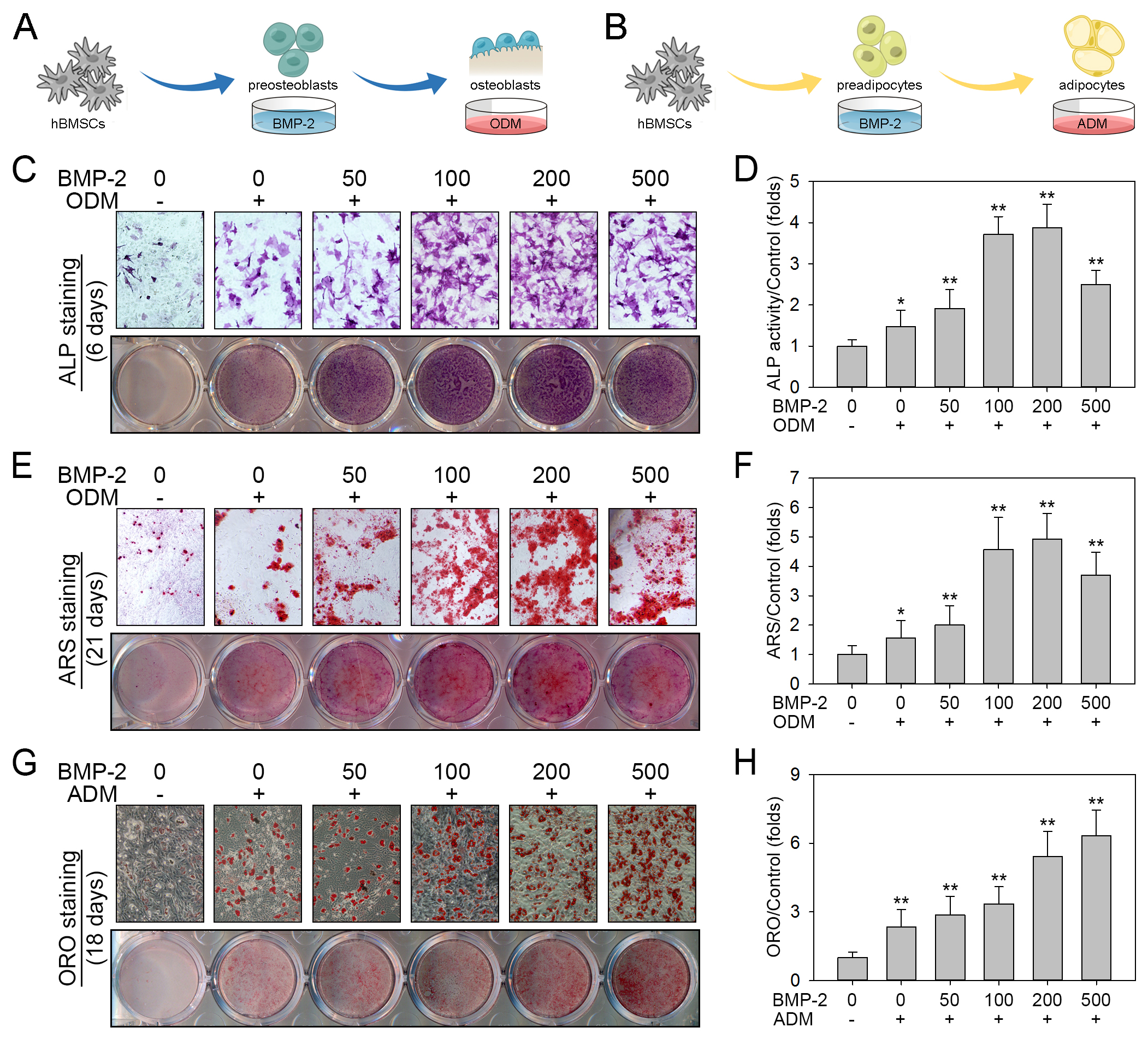

Supplement: Supplementary file 1 — Supplementary Figure 1 [file 41419_2017_126_MOESM1_ESM.jpg]

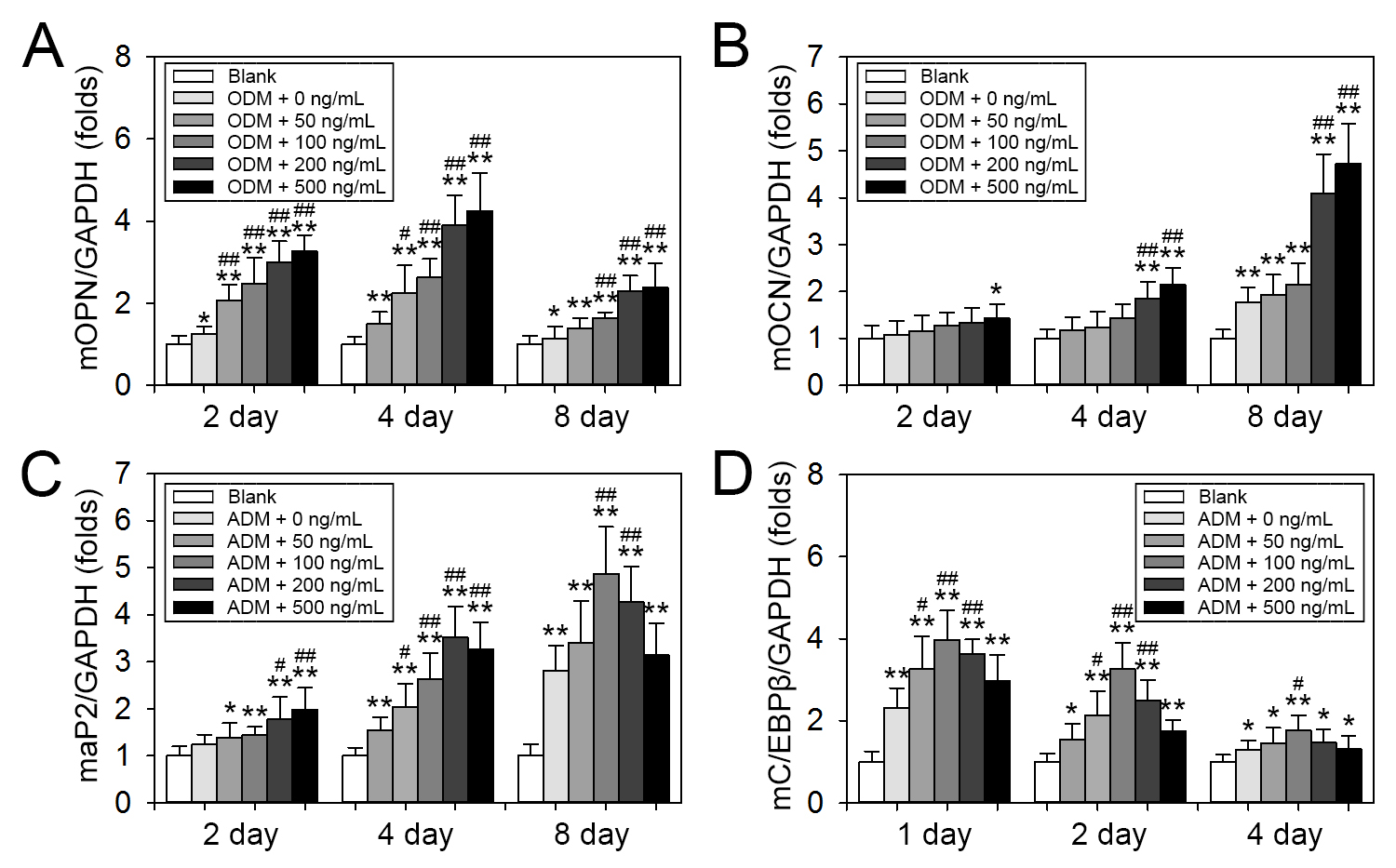

Supplement: Supplementary file 2 — Supplementary Figure 2 [file 41419_2017_126_MOESM2_ESM.jpg]
